# A prospective study on linking diarrheagenic *E. coli* with stunted childhood growth in relation to gut microbiome

**DOI:** 10.1038/s41598-023-32491-x

**Published:** 2023-04-26

**Authors:** Israr Aziz, Zobia Noreen, Umer Zeeshan Ijaz, Ozan Gundogdu, Muhammad Haroon Hamid, Nazir Muhammad, Abdullah Khan, Habib Bokhari

**Affiliations:** 1 Department of Biosciences, COMSATS University, Islamabad, Pakistan; 2 School of Engineering, University of Glasgow, Glasgow, UK; 3 Faculty of Infectious and Tropical Diseases, London School of Hygiene and Tropical Medicine, London, UK; 4 Department of Pediatrics/Department of Preventive Pediatrics, Mayo Hospital, King Edward Medical University, Lahore, Pakistan; 5 Paediatric Unit, Saidu Teaching Hospitals, Swat, Saidu Sharif, Pakistan; 6 Department of Biosciences, Kohsar University, Murree, Pakistan

**Keywords:** Clinical microbiology, High-throughput screening

## Abstract

Stunted growth is an emerging global challenge affecting children under the age of 5 years in low- and middle-income countries. Despite such a high global prevalence of stunting, the mechanism of pathogenesis and the role of associated gut microbiota is poorly understood. The present study was designed to investigate the association of pathogenic strains of *E. coli* with the residential gut microbiota of stunted growth children. A total of 64 stool sample were collected from children aged ≤ 5 years, and were processed for isolation and molecular characterization of diarrheagenic *E. coli*. Selected stool samples (n = 39 including three normal controls) were then analysed for microbial community profiling using 16S ribosomal RNA (rRNA) gene sequencing. Furthermore, associations between changes in the microbiota in the presence of different *E. coli* strains was explored. Pathotyping of the isolated *E. coli* (n = 64) has shown that 39.68% belonged to one of the five pathotypes of *E. coli* whilst the remaining ones were non-typeable. Amongst the different pathotypes, EPEC was found to be the most prevalent (52%; n = 13), followed by EAEC (20%; n = 5), EIEC (12%; n = 3), EHEC (8%; n = 2) and ETEC 2 (8%; n = 2). Phylogrouping analysis has shown that majority of the strains belonged to B2 (28.12%). Microbial diversity is shown to be significant and varied when the samples are organized under the recovered phylogroups. Moreover, based on predictive metabolism, the colonization of these strains were found to be significantly associated with energy utilization pathways such as Denovoprine-2 and glyoxylate-by. Differential analysis has shown that *Escherichia-Shigella* and *Enterococcus* were altered for the children with stunted growth.

## Introduction

Globally, approximately 180 million children under 5 years of age are affected by stunted growth, resulting in ~ 3 million child deaths annually and predominantly in low- and middle-income countries^1–3^. Stunted growth may be caused by several factors such as diarrhoea, pneumonia, and other infectious diseases; unhealthy conditions; food insecurity; and inadequate health services and care facilities^4^. In some cases, it may lead to comorbidity such as impaired cognitive development, decrease life expectancy and an increased risk of chronic diseases later in life. According to the National Nutrition Survey Pakistan (2018)^5^, four out of ten children under five years of age are stunted, whilst 17.7% suffer from wasting, 28.9% are underweight, and 9.5% are overweight. The most affected districts are in the south of the country located in the provinces of Baluchistan, Khyber Pakhtunkhwa, and Sindh, with more than 50% reported prevalence of stunting^6^. These results largely match the known socioeconomic status and district-specific multidimensional poverty indices of these regions.

Environmental Paediatric Enteropathy (EEP) is commonly associated with malnourished children and is caused due to prolonged exposure to enteric gut pathogens such as *Campylobacter* spp*, Shigella* spp, Enteroinvasive *E. coli* (EIEC), Shiga toxin-producing enterotoxigenic *E. coli* (ETEC), and typical enteropathogenic *E. coli* (tEPEC), *norovirus*, *Cryptosporidium* spp. and *Giardia* spp^7,8^. Previous studies from Pakistan have shown association of various *E. coli* pathotypes with the paediatric population showing symptoms of diarrhoea as well as mothers delivering prematurely^9–12^. Moreover, *E. coli* belonging to different phylogroup have also been associated with different clinical outcomes, and among them, phylogroup B2 had been earlier described to be the most prevalent phylogroup. It is associated with infection such as diarrhoea as it carries more virulence genes than any other phylogroup. However, the role of infection caused by *E. coli* belonging to different pathotypes and phylogroups infections at early age of life and its impact on the growth of children is still unknown.

Previous study from Pakistan highlighted the marked differences amongst rich and poor population with significantly higher prevalence of stunting in the latter^13^. In order to better understand stunted growth and avoid higher risk of mortality among young children^1^, it is essential to understand how infections due to different pathotypes and phylogroups of *E. coli* changes the microflora among stunted children which will in turn provide an insight into developing evidence based intervention approaches. Therefore, the current study investigates the different *E. coli* pathotypes and phylogroups in low socioeconomic semi-urban/urban children and their associated faecal bacterial taxa.

## Material and methods

### Ethical approval and consent

Prior written and verbal informed consent was obtained from the accompanying parent or primary caregiver of each child to participate in the study and was approved by the Ethical Review board of Department of Biosciences, CUI, Islamabad Pakistan. All methods were performed in accordance with the relevant guidelines and regulations.

### Study design & patients recruitment

The study was carried out in children (aged 1–5 years) living in Islamabad capital territories (ICT), Rawalpindi and Lahore (province-Punjab), Swat (province-KPK) and Mitthi (province-Sindh) all with the low middle income status. Children were defined as stunted if their Height-for-Age Z score (HAZ) is more than two standard deviations below the WHO child growth standards median. Additional information of study design and the criteria for patient recruitment are provided in the supplementary file (Text S1).

### Faecal sample collection

A total of 64 stool samples (60 stunted and 4 normal; morning first bowel from children at hospital) were collected using commercially available uBiome microbiome sampling kits (uBiome, USA). These kits follow the protocols outlined by the NIH Human Microbiome Project^14^. One sterile swab was used to transfer a small amount of faecal material into a vial containing a lysis and stabilization buffer that preserves the DNA for transport at ambient temperatures. The other sterile cotton swab was used to collect the faecal material to be shipped in transport media for culturing. The stool samples from the transport media were then processed by streaking on MacConkey agar (Oxoid) and incubated at 37 °C overnight. Pure cultures were obtained by re-culture of grown colonies for another 18–24 h at the same incubation temperature. *E. coli* was further confirmed using the standard biochemical tests including catalase, oxidase, and indole tests.

### DNA extraction & gene specific PCR for *E. coli* pathotypes and phylogroup screening

Genomic DNA was extracted by ethanol precipitation technique as described previously^13^. The extracted DNA was used for polymerase chain reaction (PCR) to screen the isolates for the presence of pathotype using gene specific primers in two-step multiplex PCR. Briefly, nine primer pairs were used, the first set comprised of four primers, including *eae, bfp, vt* and *aggR* for EPEC, EAEC, and EHEC, whilst the second set consisted of five primer pairs targeting the genes *st, lt, virF, ipaH* and *daaE* as explained previously^9^. Phylogenetic grouping of *E. coli* was done using the revised quadruplex PCR method described by Clermont et al. 2013^15^. Each isolate was assigned one of the eight phylogenetic groups (A, B1, B2, C, D, E, F, Clade I) by targeting three marker genes (*ArpA, ChuA, YjaA*) and a DNA fragment TSPE4.C2 (Supplementary Table 1). Plasmids containing target genes (gift from Oscar G. Gomez-Duarte, International Enteric Vaccines Research Program [IEVRP], University of Iowa Children's Hospital, Iowa City, IA) were used as positive controls and the DNA from *E. coli* DH5α was used as a negative control.

### Microbiome analysis-I6S rRNA sequencing-microbial taxa

For microbiome studies, random 39 stool samples from which *E. coli* belonging to different phylogroups were processed and these steps were performed in the CLIA-compliant (Clinical Laboratory Improvement Amendments) and CAP-accredited (College of American Pathologists) uBiome laboratory in San Francisco, CA, USA. Samples were lysed using mechanical bead-beating. DNA was extracted and purified by a liquid- handling robot in a class 100 clean room using the method described previously^16^. The V4 region of 16S rRNA gene was PCR amplified using universal forward and reverse primers (515F: GTGCCAGCMGCCGCGGTAA and 806R: GGACTACHVGGGTWTCTAAT). Primers also contained Illumina tags and barcodes with unique combination of forward and reverse indexes to allow multiplexing. Pooled PCR products were column-purified and selected through microfluidic DNA fractionation based on size^17^. Real-time qPCR quantified consolidated libraries using the Kapa Bio-Rad iCycler qPCR kit on a BioRad MyiQ prior to sequencing.

### Bioinformatics and statistical analysis

Bioinformatics methods were based on Ijaz et al. 2018 with minor modifications^18^. The VSEARCH v2.3.4 pipeline was used to produce the abundance table by constructing operational taxonomic units (OTUs), a representation of species, as described in http://github.com/torognes/vsearch/wiki/VSEARCH-pipeline). For detailed bioinformatics and statistical analysis, supplementary files are provided in Supplementary_Materials.docx along with meta data information including location and demographic data given in Supplementary_Metadata.xlsx.

## Results

### Culturing and isolation

*E. coli* were isolated from all 64 collected stool samples and were initially screened using standard gram staining and biochemical tests including catalase, oxidase and indole tests and all isolates were found to be positive for *E. coli*.

### Recovered *E. coli* pathotypes

**Figure 1:**
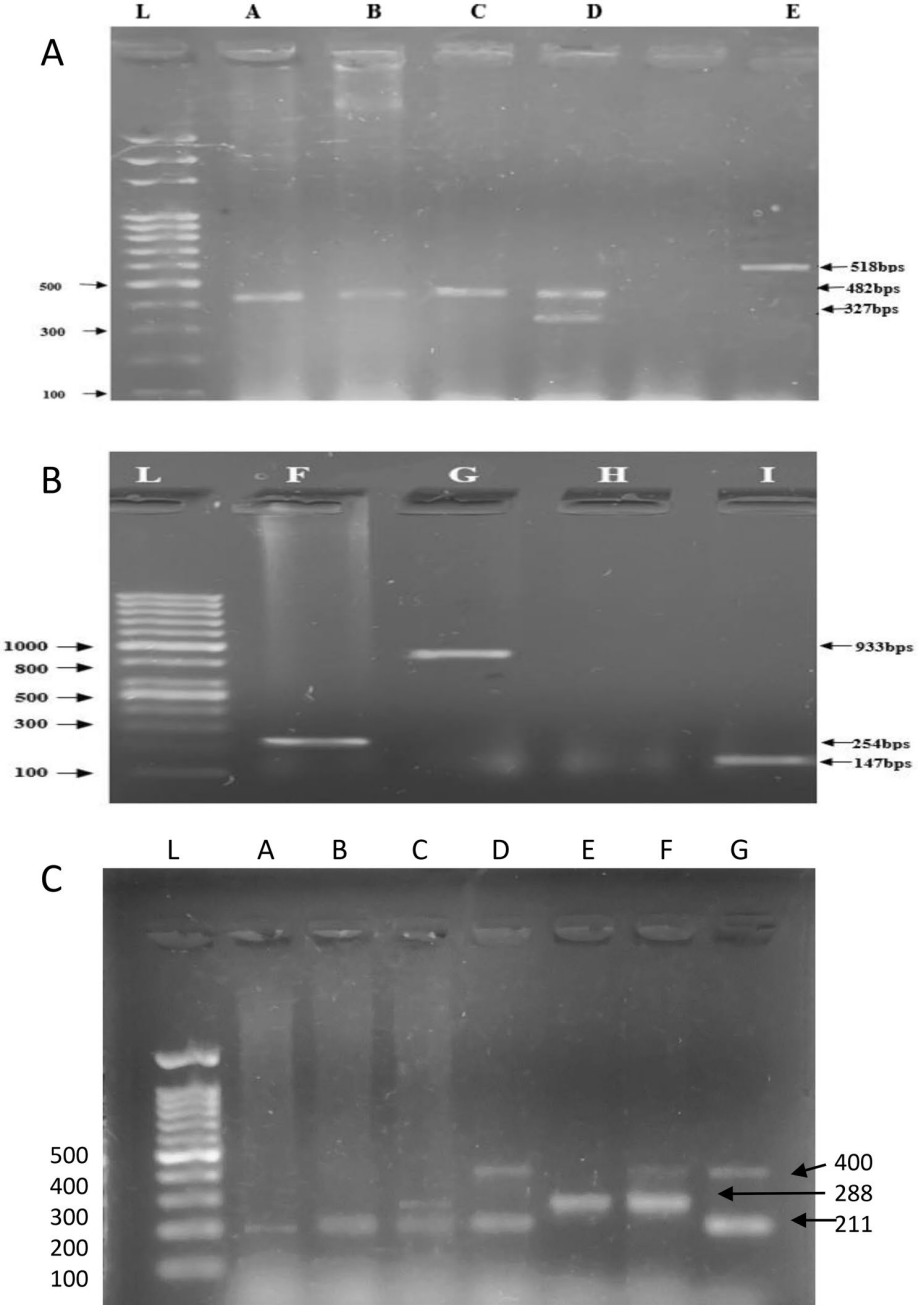
Molecular characterization of *E. coli* pathotypes and phylogroups**.** (**A**) 2% Agarose gel presenting amplified products of multiplex PCR for pathotyping. L represents ladder while lane A, B and C shows the band of eae corresponding to aEPEC while lane D shows the band of eae as well as bfp corresponding to typical EPEC. E Lane presents the band of vt corresponding to EHEC. (**B**) L represents ladder and lane F shows band for aggR corresponding to EAEC while lane G shows the band of ipaH corresponding to EIEC, and lane I shows band st corresponding to ETEC. (**C**) 2% Gel image showing Agarose gel presenting amplified products of multiplex PCR for phylotyping combination of four different amplicons, ArpA, chuA, yjaA TSPE4.C2, having 400bps, 288bps, 211bps, 152bps size, representing *E. coli* different phylogroups isolated from stunted growth children. First lane L shows 100 bp molecular weight ladder, lane A & B shows bands for *E. coli* clade 1, C lane indicates B2, lane D indicates C phylogroup while lane E presents F phylogroup and F lane shows E phylogroup.

The results suggested that out of a total of 64 samples, 25 (39.68%) were positive for any five out of the six diarrheagenic *E. coli* pathotypes whereas 39 samples (60.9%) were non-typeable (NT). Within the diarrheagenic pathotypes, the highest ratio of EPEC 13 (52%) was observed, and this was followed by EAEC 5 (20%), EIEC 3 (12%), EHEC 2 (8%) and ETEC 2 (8%) as shown in Fig. 1A,B.

### Recovered *E. coli* phylogroups

Amongst the isolated *E. coli* strains from 64 samples, 18 of them belonged to B2 (28.12%), 8 belonged to *E. coli* Clade 1 (12.50%), 8 belonged to A (12.5%), 7 belonged F (10.93%), 6 belonged to E (9.37%), whereas 4 belonged to C (6.25%), and 2 belonged to D (3.12%). 12 of them were non-typeable (18.75%) and none of the isolates belonged to B1 as illustrated in Fig. 1C.

### Diversity patterns representative of pathotypes and phylogroups

**Figure 2:**
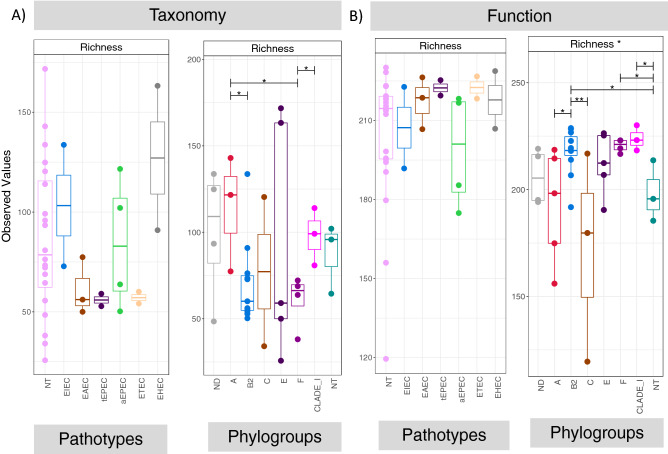
Rarefied Richness estimates for OTU abundance table (**A**), and for recovered MetaCyc pathways (**B**), with samples organized under a given convention, whether it is pathotypes or phylogroups. Two groups are connected together with a line if the ANOVA values are significant.

Microbial diversity of stunted individuals (n = 36) for different pathotypes and phylogroups (both in terms of taxonomy using OTUs and function using MetaCyc pathways recovered from PICRUSt2) was estimated using rarefied richness estimates. There was no significant difference in richness when the stunted samples were organized according to pathotypes. However, when the samples were organised under phylogroups, significant differences in richness were observed during pair-wise comparisons. This affected both taxonomy/composition (between A and B2; A and F; Clade-1 and F) and their recovered metabolic functions (between A and B2; B2 and C; B2 and NT; F and NT; and Clade-1 and NT). Highest richness in terms of taxonomy/composition was observed for phylogroups E and A, whilst phylogroup Clade-1 have shown maximum richness in terms of recovered metabolic functions (Fig. 2).

**Figure 3:**
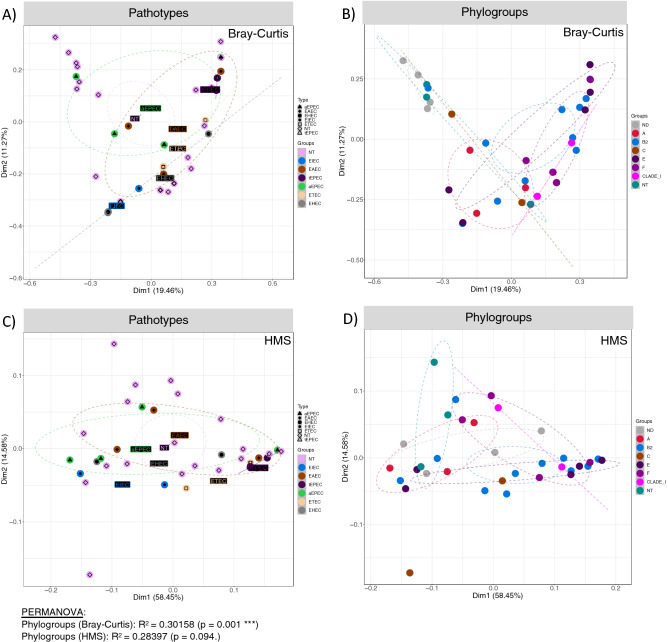
Principal coordinate analysis (PCoA) using different several dissimilarity indices [Bray–Curtis distance and hierarchical meta-storm (HMS)] where ellipses were drawn using 95% confidence intervals based on the standard error of ordination points for a given category. Beneath each figure are the R^2^ values (along with p-values if significant) calculated from PERMANOVA.

Beta diversity among different groups were analysed using different dissimilarity indices i.e. Bray–Curtis (for composition) and Hierarchical Meta-storm (HMS; for function). No significant difference in both was observed among pathotypes (Fig. 3A,C). On the other hand, beta diversity varied significantly (PERMANOVA) between different phylogroup for both composition (30% variability explained; p = 0.001) and function (28% variability explained; p = 0.094), respectively (Fig. 3B,D).

**Figure 4:**
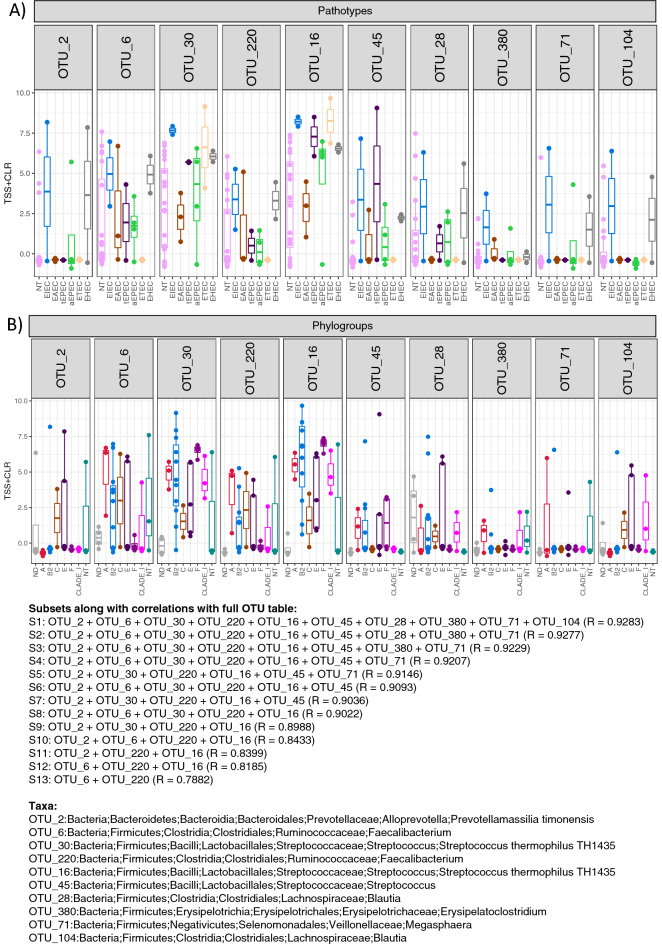
Expression of significant OTUs identified from the BVSTEP routine with samples organized under a given convention, whether it is pathotypes (**A**) or phylogroups (**B**). The information, and the taxonomy of OTUs from the top subsets is given below the figure with the correlation between Bray–Curtis distances between a particular subset and the full OTU table given in parenthesis.

**Figure 5:**
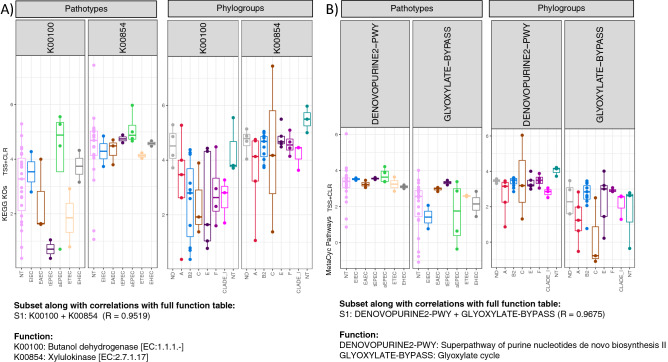
Expression of significant KEGG Orthologs KOs (**A**), and MetaCyc pathways (**B**) identified from the BVSTEP routine with samples organized under a given convention, whether it is pathotypes or phylogroups. For the given data, only one subset was returned with > 0.95 correlation with further details including correlation values given below the figures.

To identify the key drivers which result in the changes in beta diversity among the samples, the ‘BVSTEP’ routine was used. The method conserves beta diversity patterns between the samples whilst reducing the feature space, whether it is OTUs or pathways (KEGG KOs and MetaCyc pathways), by following a permutation approach that iterates through subset of features. The method also uses a quality-of-fit criteria, correlation coefficient R, to assess how much beta diversity is conserved between samples when considering a select subset of features. The resulting highly variable OTUs belonged to the genera *Prevotella*, *Faecalibacterium*, *Streptococcus*, *Blautia*, *Erysipelatoclostridium* and *Megasphaera*, and were increased in the EIEC Group. *Prevotella*, *Faecalibacterium*, *Blautia*, *Erysipelatoclostridium* and *Megasphaera* were found to be increase in EHEC group as compared to the other groups. Also, *Streptococcus* and *Erysipelatoclostridium* were found to be the dominant genera of OTUs in ETEC and EAEC group, respectively (Fig. 4A). In terms of organisation under phylogroups, OTUs belonging to the genera *Prevotella* and *Blautia* were more abundant in phylogroup E, with Faecalibacterium*, Erysipelatoclostridium* and *Megasphaera* comparatively more abundant in phylogroup A. In phylogroup B2 and F, OTUs of *Streptococcus* were more abundant as compared to the other groups (Fig. 4B). After applying BVSTEP routine to the PICRUSt2 tables, and irrespective of sample organization used whether pathotypes or phylogroups, we did not find much variation, and only a subset comprising KEGG KOs K00100 and K00854 (encoding for butanol dehydrogenase and Xylulokinase) to have variation between the samples. (Fig. 5A). Similarly, two MetaCyc pathways (DENOVOPURINE2-PWY and GLYOXYLATE-BYPASS) encoding denovopurine-2 and glyoxylate bypass was found to be the main source of variability amongst the samples (Fig. 5B).

### Regression analyses using generalised linear latent variable model (GLLVM) procedure

**Figure 6:**
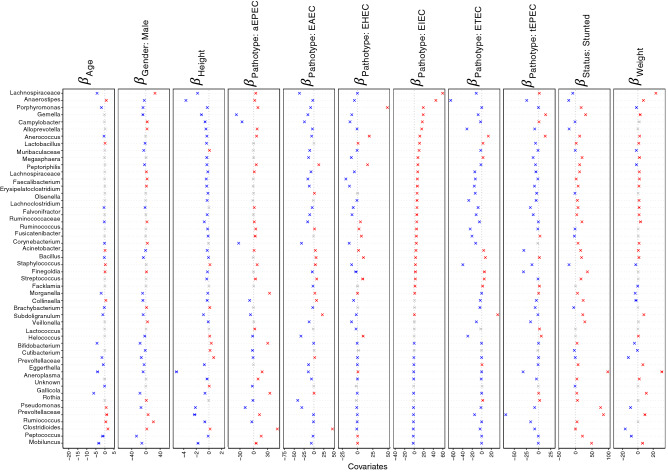
Beta-coefficients returned from the GLLVM procedure for covariates considered in this study by considering top 100 most abundant genera incorporating continuous data (Age, Height, and Weight) as well as categorical labelling of samples (Gender, Male, and Pathotypes). Those coefficients which are positively associated with the microbial abundance of a particular species are represented in red colour whilst those that are negatively associated are represented with blue colour, respectively. Where the 95% confidence interval of the beta-coefficients crosses the 0 boundary, i.e., they are insignificant, are represented by grey colour. Since the collation of the OTUs were done at Genus level, all those OTUs that cannot be categorized based on taxonomy are collated under “Unknown” category. The figure only shows beta-coefficients for 48 genera with the remaining ones shown in Fig. 7.

**Figure 7:**
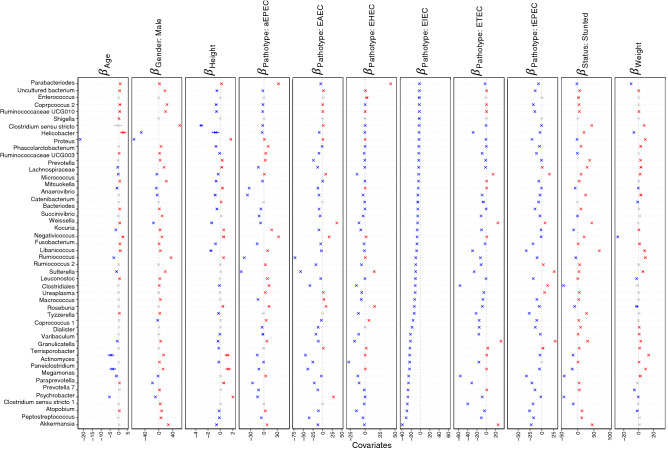
Continuation of Fig. 6 for the remaining beta-coefficients for 52 genera returned from the GLLVM procedure when considering pathotypes in the analysis.

**Figure 8:**
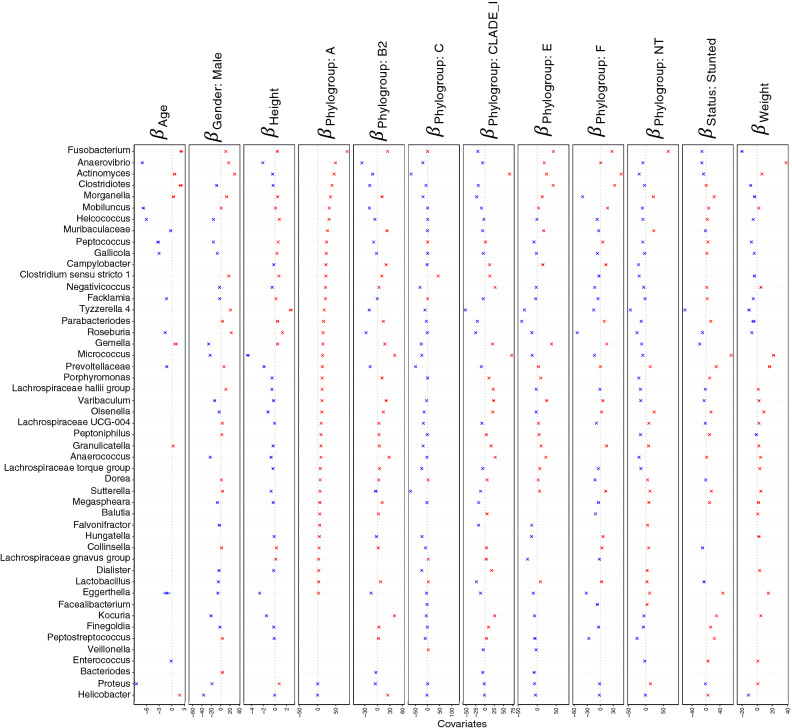
Beta-coefficients returned from the GLLVM procedure for covariates considered in this study by considering top 100 most abundant genera incorporating continuous data (Age, Height, and Weight) as well as categorical labelling of samples (Gender, Male, and Phylogroups). Those coefficients which are positively associated with the microbial abundance of a particular species are represented in red colour whilst those that are negatively associated are represented with blue colour, respectively. Where the 95% confidence interval of the beta-coefficients crosses the 0 boundary, i.e., they are insignificant, are represented by grey colour. Since the collation of the OTUs were done at Genus level, all those OTUs that cannot be categorized based on taxonomy are collated under “Unknown” category. The figure only shows beta-coefficients for 49 genera with the remaining ones shown in Fig. 9.

**Figure 9:**
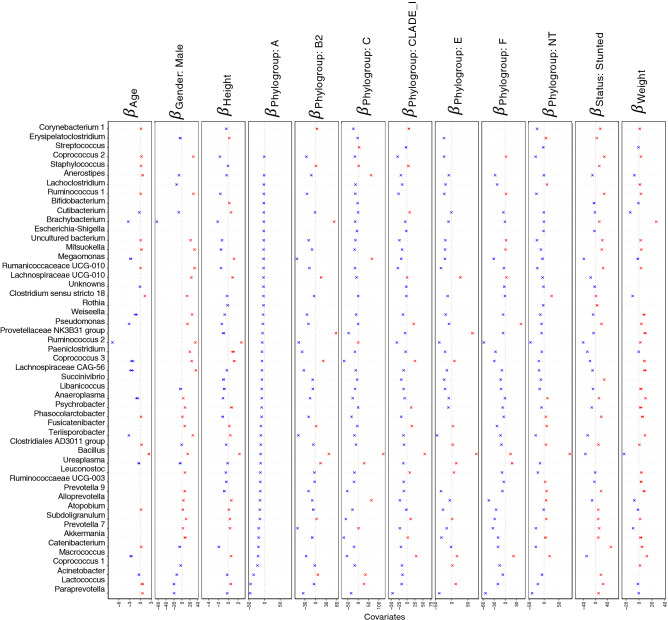
Continuation of Fig. 8 for the remaining beta-coefficients for 51 genera returned from the GLLVM procedure when considering phylogroups in the analysis.

We ran two different models in GLLVM to associate microbial abundance of top 100 genera with the environmental covariates (Age, Height, and Weight, Gender, pathotypes, phylogroups and Stunting Status), one for samples characterised under pathotypes and one for phylogroups, respectively (Figs. 6, 7, 8 and 9). Here, we have also included the normal samples as a reference (n = 39 with 36 stunted and 3 normal samples). Recovery of beta coefficients on the basis of pathotypes have shown that *Anaerostipes* and *Libanicoccus* were negatively associated with height and positively associated with the stunting status. Weight was positively associated with the beneficial flora belonging to *Lachnospiracae*, *Weissella* and *Clostridium *sensu stricto, whilst negative associations were observed for *Paraprevotella*, *Ruminococcus*, *Lachnospiraceae* (Figs. 6 and 7).

Analysis on the basis of phylogroups have shown that the males showed positive association with normal flora belonging to *Actinomyces*, *Ruminococcaceae*, and *Lachnospiraceae*. Height was positively associated with *Tyzzerella* and negatively associated with stunting. However, positive association of *Eggerthella* was observed for stunting. Whilst all the phylogroups have shown negative association with the bacteria belonging to normal flora of human gut, B2 phylogroup has shown a pronounced negative association with majority of normal flora, i.e., *Anaerovibrio*, *Ruminococcus*, *Paeniclostridium*, *Lachnospiraceae*, *Terrisporobacter Baciilus* and *Prevotella* (Figs. 8 and 9).

### Quantifying spatial structuring mechanisms in assemblages of microbial communities of stunted individuals

**Figure 10:**
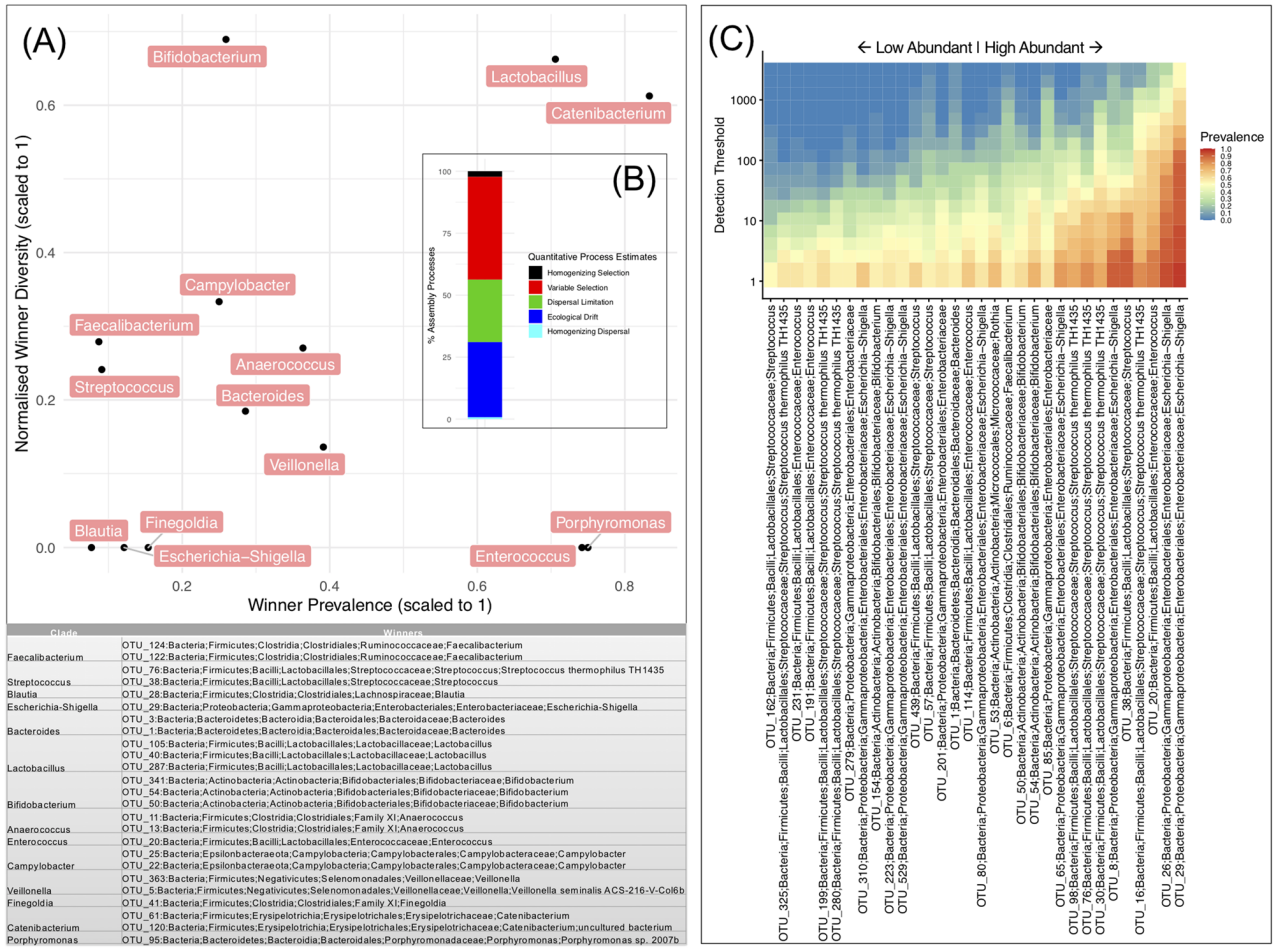
Community structure of samples with “stunted” status. (**A**) Competitive lottery model for conceptualising community assembly of these samples with genera displaying lottery-like behaviour plotted by prevalence and diversity of the ‘winning’ OTUs; these ‘winning’ OTUs (> 90% abundance for that clade) from each clade are given in the bottom table. The inset (**B**) gives the assembly processes using QPE estimates. (**C**) Core microbiome of samples with “stunted” status where we have used a minimum prevalence of 50% of OTUs in these samples as the lower limit.

Lottery model for clade-based assembly were used with the underlying premise being that within a clade, closely related species will exhibit a very strong degree of competition i.e., of all the members in a given clade, the fittest member (or the first member to colonize) will become the most abundant and will dictate the community structure (Fig. 10). These clades were defined taxonomically within each genus. From these clades, lottery ‘winners’ were identified as those species which have > 90% abundance within each clade, with the x-axis representing lottery type clade prevalence as a fraction of the total number of samples, whilst the y-axis represents the diversity if multiple species within a clades are lottery winners. For clades where there is only one lottery winner, the winner diversity values go to zero. The bottom figure (Fig. 10A) shows the winners identified in each clade. The strongest lottery-like groups however have a high diversity and high winner prevalence (*Lactobacillus* and *Catenibacterium*) and indicate a more variable and random (stochastic) selection of OTUs as winners within the clade. This may also provide insights into how “stunting microbiome” is shaped. Such winners can be thought of as strong competitors regardless of disease aetiology and are more resilient. Conversely, the lottery-type clades at the bottom always select the same species, suggesting their link to stunting (can be deemed as signature species to stunting). *Escherichia-shigella*, *Enterococcus* and *Porphyromonas* are the prevalence winners and shows a strong link with stunting. The inset (Fig. 10B) is the overall assembly processes breaking down the community assembly mechanisms into the ecological processes using QPE estimates. QPE estimates revealed that the amount of stochasticity (Dispersal limitation, Homogenizing dispersal, and ecological drift explain roughly 55% of community assembly, whilst amongst the deterministic causes (*Homogenizing Selection*, and *Variable Selection*), high variable selection implies multiple stable community structures possible in stunted individuals. We then calculated the core microbiome (with 50% minimum prevalence) to identify the signature species associated with stunting. Figure 10C shows that taxa on the right side of the heatmap are not only highly prevalent but also have very high abundances. These taxa include *Escherichia–Shigella*, *Streptococci* and *Enterococci.*

Other than finding marginal changes in microbiota associated with Gentamicin usage and whether the child was breastfed at birth, we did not find any other observed clinical covariates explaining variability in microbial community structure (Supplementary Table 2).

## Discussion

Environmental Paediatric Enteropathy (EEP) is widespread among malnourished children and has been associated with prolonged exposure to enteric gut pathogens including *Campylobacter species, Shigella spp*, enteroinvasive *E. coli*, heat-stable toxin-producing enterotoxigenic *E. coli*, shiga toxin-producing enterotoxigenic *E. coli* and typical enteropathogenic *E. coli,* norovirus, *Cryptosporidium spp*. and *Giardia spp* leading to stunting at early age^19–22^. This study was designed to explore the effect of exposures of different pathogenic strains of *E. coli* at early age of life and their impact on microbiota in low socioeconomic semi-urban/urban setting.

As majority of the *E*. *coli* strains in human gut are part of normal microbiota, infection due to pathogenic strains of *E. coli* can be determined by the presence of virulence genes. The pathogenic strains can be classified either on the basis of pathotypes or phylogroups^9,15^. Pathotypes of diarrheagenic *E. coli* differ from each other in terms of virulence genes and how they interact with eukaryotic cells. Previous studies from Pakistan have shown association of EPEC and EAEC pathotypes with the paediatric population and preterm mothers^9–13^. In this present study, *E. coli* were isolated from the gut of asymptomatic children showing stunted growth and were further screened for pathotypes distribution and associated bacterial taxa, and were compared against a normal cohort. The results suggested that a total of 25 (39.06%) were positive for five of diarrheagenic *E. coli* pathotypes, presenting EPEC 13 (52%) as a dominant pathotype followed by EAEC 5 (20%), EIEC 3(12%), EHEC 2(8%), and ETEC 2(8%), respectively. Our study corroborates with similar study carried out in India, Kenya, Tehran where EPEC was found as the dominant pathotype in children under 5-years of age^23–25^.

Phylogroup characterization of gut *E. coli* provides insight into the genetic structure of the phylogroups (A, B1, B2, C, D, E, and F) which differ in their phenotypic traits, ecological niche and virulence potential^15^. In this study, the phylogrouping analysis has shown that majority of the *E. coli* strains isolated from stunted growth children belonged to Phylogroup B (28.12%). Phylogroup B2 had been earlier described to be the most prevalent phylogroup associated with infection such as diarrhoea, urinary tract infection and colorectal neoplasm, as it carries more virulence genes that any other group^26–28^. Moreover, B2 has the ability to persist for longer periods in infants than other *E. coli* strains and therefore can influence the microbiota^29^. Therefore, the association of B2 phylogroup with stunted growth in our study might provide a glimpse of associated illness that might lead to stunting.

The colonization of enteropathogens subsequently results in reduced diversity and abundance of useful gut microbial flora negatively influencing the functions of gut^19,30–32^ by hampering production of important vitamins, antioxidants as well as hindering the complete digestion of food resulting in further loss of 10–15% nutrition as it passes unused through the digestive system^33^. In this study, we analysed the effect of colonization of different pathotypes and phylogroups of *E. coli* on the gut microbial diversity, composition and functional makeup in stunted growth children. After identification of pathotypes and phylogroups, a subset of 39 faecal samples were sequenced and analysed for gut microbiome analysis. Both taxonomic and functional diversity were different between the recovered phylogroups of *E. coli* suggesting that different phylogroups have varying ability to compete for niche and nutrients. The taxonomic richness was found to be lower in samples where Phylogroup B2 and F colonized. However, highest functional richness was found to be in B2 phylogroup which we hypothesize is due to enhance competitive nature of the strains^34^. This results in alternative pathway to avail nutrient and energy. Diversity patterns were differentially more pronounced when samples were categorized under phylogroups as compared to when they were categorized under pathotypes.

Majority of the key players to vary significantly among samples are strict anaerobes suggesting that colonization of *E. coli* phylogroups alter the gut niche. In terms of function, Key KEGG orthologs which varied significantly encoded for butanol dehydrogenase and Xylulokinase. Butanol dehydrogenase (K00100) uses butanol as a source of carbon for energy production in nutrient depleted niche and also helps protect against anaerobic stress^35^. Xylulokinase (K00854) can convert xylose, a pentose, to xylulose 5-phosphate and is also a player in energy production^36^. Denovoprine-2 and glyoxylate bypass were found to be the two key pathways which varied significantly between the phylogroups. Denovoprine-2 is used in nucleotide biosynthesis process resulting in more energy generation for enhanced colonization and virulence^37^. Glyoxylate bypass or shunt also varied significantly among is used by microbes when they are in nutrient stress to conserve carbon atoms for gluconeogenesis and promote energy production^38^. Overall colonization of different phylogroups affect changes in carbohydrate metabolism, and nucleotide synthesis which might be attributed to the competitive nature of the strains.

To further investigate this, GLLVM analysis was carried out by including normal cohort. Colonization of pathotypes were negatively associated with the normal microflora (*Paraprevotella, Ruminococcus, Lachnospiraceae*) indicating that these strains reduced the beneficial bacteria in the gut which might have led to stunting. Analysis on the basis of phylogroups highlighted height to be positively associated with *Tyzzerella* and negatively associated with stunting, thus indicating its potential role in normal growth and development^39^. Among the pathotypes recovered, colonization of B2 phylogroup has shown the most drastic effect on normal microflora as indicated by a negative association with normal flora belonging to genera *Anaerovibrio*, *Ruminococcus*, *Paeniclostridium*, *Lachnospiraceae*, *Terrisporobacter*, *Baciilus* and *Prevotella*.

Core microbiome analysis for clade-based assembly has shown that *Escherichia-shigella*, *Enterococcus* and *Porphyromonas* are more prevalent and strongly linked with stunting. This is in line with previous studies which have shown that *Escherichia-shigella*, *Streptococcus* and *Lactobacillus* are associated with stunted growth in children^39^. Our study has shown that colonization of different phylogroups of *E. coli* (especially B2) significantly changed the intestinal microbiota of stunted growth children. This leads to the conclusion that a disruption in the equilibrium between the bacterial EED aetiology and co-residing bacterial communities might well be linked with subsequent poor growth performance. Furthermore, investigation involving a larger cohort and inclusion of sizeable control group is desirable, as these were the major limitations of the present study.

## Conclusions

The present study shows that exposure to pathogenic strains of *E. coli* drastically changes the gut microbiota of stunted growth children leading to reduction in beneficial microbiota and altered microbial diversity. E coli strains belonging to phylogroup B2 by virtue of their competitive nature had a profound effect on the gut microbial ecology as compared to strains belonging to other phylogroups and pathotypes. Understanding the vulnerable child gut microbiome in relation to the exposome is a route to develop suitable intervention strategies to reduce the burden of EED aetiologies and hence interlinked stunting.

## Supplementary Information

Supplementary Information 1.

Supplementary Information 2.

## Data Availability

The dataset presented in this study is available under ENA repository PRJEB58411 with the meta data provided in the supplementary material.
