# Supplementary material for: A prospective study on linking diarrheagenic E. coli with stunted childhood growth in relation to gut microbiome
Source: Sci Rep. 2023 Apr 26;13:6802. doi: 10.1038/s41598-023-32491-x (PMC10133260; doi:10.1038/s41598-023-32491-x)
Supplement: Supplementary file 1 — Supplementary Information 1. [file 41598_2023_32491_MOESM1_ESM.docx]

**Study Design & Patients Recruitment**

Household questionnaire on demographics; socioeconomic status (livelihoods/wealth); maternal health, animal and backyard poultry ownership their management, housing, water quality and sanitation and hygiene conditions were filled in an interview with the accompanying parent visiting the Hospital at the time of enrolment. Moreover, short questionnaire on child health, vaccinations, breastfeeding practices, antibiotic use, and diet was also collected as well as while collecting fecal samples. The study protocol was approved by the ethical review boards of COMSATS University, Islamabad, associated hospitals (Mayo Hospital, Lahore and PIMS, Islamabad). The inclusion and exclusion criteria for recruiting were adopted as described from previous study briefly the inclusion criteria include i) children under 5 years of age and capable of participating in the different tests and clinical sampling; ii) exclusion criteria include severe acute illness, respiratory distress, fever, HIV-associated enteropathy or severe diarrhoea, vomiting septic shock and recent antibiotic treatment or nutrition regimens [1]. The nutritional status of children was measured by its Z-score and falls in the range of -2 to -4, which incorporate anthropometric measurements of height/length of height/weight, according to the child Growth Standards of WHO. Briefly, children were grouped, as per median height of the WHO reference population into three categories: extreme stunting (height for-age z-score ≤ −3 SD), moderate stunting (height-for-age z-score between −3 SD and −2 SD), and not stunted (height-for-age z-score ≥ −2 SD) [2-4]. In more simplified terms, children were considered stunted (based on anthropometric measurements of height/length of height/weight) e.g. if their height-for-age is greater from two times standard deviations below the World Health Organization (WHO) Child Growth Standards median <https://www.who.int/childgrowth/standards/en/>.

**Bioinformatics**

Briefly, paired-end reads were pre-processed based on guidelines from recent using a sliding window approach) and reads were filtered using Sickle v1.33 [5]. BayesHammer [6] was used from Spades v3.1.1 assembler to perform error-correction on paired-end reads. Then, PANDAseq (v2.11) [7] was used to assemble the forward and reverse reads into a single sequence spanning the entire V4 region with a minimum overlap of 50 bp. Reads were then pooled, dereplicated, sorted in order of decreasing abundance and singletons were discarded. Following this, the reads were clustered based on 97% similarity and had removal of clusters performed with chimeric models built from more abundant reads (-- uchime_denovo option in vsearch). In addition, a reference-based chimera filtering step (--uchime_ref option in vsearch) was performed using a gold database (https://www.mothur.org/w/images/f/f1/Silva.gold.bacteria.zip). Finally, the OTU table was generated by matching the original barcoded reads against clean OTUs. The representative OTU sequences were then loaded into Qiime2 [8] followed by assigning a taxonomy to represent the OTUs against the SILVA SSU Ref NR database release v132 database [9]. A phylogenetic tree within Qiime2 was created with align-to-tree- mafft-fasttree using MAFFT (v7.310) [10] and FastTree (v2.1.10) [11]. A BIOM file was created within Qiime2 merging the otu_table.txt (abundance table) and the taxonomy.tsv (taxonomy) for compatibility to R and phyloseq [12]. This gave an OTU abundance table with n=38 samples, and P=834 OTUs with summary statistics of samples as follows: [1^st^ Quartile: 27,113; Median:52,190; Mean: 53,511; 3^rd^ Quartile:71,080; Maximum: 136,425].

The PICRUSt2 QIIME2 plugin was used to predict functional abundance and diversity in the form of KEGG enzymes (KEGG Orthologs [KOs]) and MetaCyc pathways. The algorithm uses reference genomes for which KEGG KOs are available, and it then checks individual OTUs by using maximum Nearest Sequenced Taxon Index (NSTI) cut-off of 2.0 (a measure of closeness to the reference genome with values < 2.0 sufficient to obtain a reference hit). Because PICRUSt2 has a comprehensive database (~20,000 genomes) as compared to its predecessor and that a very strong correlation (consistently > 0.8) between the actual functions obtained through shotgun metagenomics and those predicted by PICRUSt2 is reported in literature [13], therefore, it increases our confidence in the functions recovered.  In total, for 38 samples, we recovered 9,378 KOs, and 461 MetaCyc pathways.

**Statistical Analysis**

For community analysis (including alpha and beta diversity analyses) we used the vegan package [14]. For alpha diversity metrics, *Rarefied Richness* is an estimated number of species/features per sample after rarefying for minimum library size. Principal Coordinate Analysis (PCoA) plots of OTUs using two different distance measures in Vegan’s cmdscale() function: *Bray-Curtis,* which is a distance metric that considers only OTUs abundance counts; and *Hierarchical Meta-Storms* (HMS) [15], a new function beta diversity measure that relies on KEGG KOs. The reason for choosing HMS over Bray-Surtis distance is as follows. Although it is straightforward to calculate taxonomic beta diversity by assuming independence between features (each microbe/OTU is a distinct entity) and traditionally Bray-Curtis distance (or any other count measure) are also applied on functional abundance (KEGG Orthologs) tables obtained from PICRUSt2, the returned functional dissimilarities between the samples are not accurate and result in erroneous distances. This is mainly because there is redundancy in KOs, with functional pathways (that build on these KOs) typically able to utilise alternative KOs in enzymatic reactions. To capture the hierarchy and inherent dependences between KOs, HMS collapses the metabolic pathways at the observed KOs level by considering BRITE pathways as a set of reference pathways, and then propagates the abundances upward for these pathways in a multi-level pathway hierarchy to give a weighted dissimilarity measure. This then provides higher sensitivity for detecting variations in upper-level metabolic pathways between samples. Analysis of variance was performed using Vegan’s adonis() with distance matrices (Bray-Curtis/HMS) against sources of variation, and are reported on beta diversity plots. This function, referred to as PERMANOVA, fits linear models to distance matrices and used a permutation test with pseudo-F ratios and gives percentage variability in microbial community explained by a particular covariate as R^2^ value (if significant).

The community assembly processes were also assessed using the quantitative process estimates (QPE) method [16]. This is based on an ecological framework that describes assembly processes in terms of selection (variable or homogenous), dispersal (dispersal limitation, or homogenizing dispersal (mass effects) or random drift. The method breaks down assembly processes in terms of selection (variable or homogenous), dispersal (dispersal limitation, or homogenising dispersal) and undominated/ecological drift (that cannot be explained) mechanisms. Variable selection gives rise to high compositional differences in community structure due to multiple environmental conditions, whilst homogenous selection occurs when consistent environmental conditions result in consistent pressure. Dispersal processes refer to the movement of microbes throughout space, whether they are absent (dispersal limitation) or present with high rates (homogenising dispersal) resulting in homogenisation. For human associated microbiome studies, dispersal limitation is typically expected (as opposed to environmental studies). The method uses deviation from the observed βMNTD (β-mean-nearest-taxon-distance) and the mean of the null distribution was evaluated using βNTI (β-nearest-taxon-index). When the observed value of βMNTD deviated significantly from the null expectation, the community is assembled by variable (βNTI >+2) or homogenous (βNTI < −2) selection processes. If the difference is not significant, the observed differences in phylogenetic composition are considered to be the result of dispersal mechanisms enabling ecological drift. These are differentiated using the abundance-based β_RC_ and a Bray-Curtis dissimilarity metric for beta diversity. If the β_RCbray_ > + 0.95, assembly is explained by dispersal limitation; if β_RCbray_ < −0.95 then homogenising dispersal mechanisms contribute to community assembly; and if β_RCbray_ was between −0.95 and +0.95, community turnover is due to undominated mechanisms/ecological drift, i.e., neither explained by dispersal nor explained by selection processes.

Taking an altogether different approach to conceptualize community assembly, the lottery model for clade-based assembly assumes that within a defined clade, species will exhibit a high degree of competition. The most fit member, or simply the first member to arrive (exhibiting strong priority effects) will become the most abundant and will dictate the shape the community takes henceforth. Clades were defined taxonomically at the genus level – assuming that within each genus, species would exhibit strong degrees of phylogenetic similarity (similar gene content, metabolic capabilities, niche preferences, etc.) resulting in intense within-group competition. From these clades, lottery ‘winners’ were identified. Winners were defined as achieving >90% abundance within the defined clade (threshold choice based on recommendations [17] after performing simulations based on stick-breaking models). These winners were categorized based on winner prevalence (the fraction of samples which included a winner OTU for that family) and winner diversity (the frequency that each OTU occurs as the winner in the samples from which winners were observed). Strong lottery behavior is exhibited when both diversity and prevalence are high, indicating that nearly all the samples had winners within the genera (prevalence) and that each OTU had an even chance to be the winner (diversity). Genera from across the phylogenetic tree were deemed to have winning members, although several clades did not display any lottery- like behavior.

The “BVSTEP” routine [18] was used for subset analysis, a procedure through which the abundance table is imploded down to minimum set of features in such a way that the reduced table preserves roughly the same beta diversity (Bray-Curtis distance) between samples as the full abundance table, choosing those subsets that have the highest correlation. To run this algorithm, bvStep() (from the sinkr package) [19].

To find the relationship between microbial communities and sources of variation, we have used Generalised Linear Latent Variable Model (GLLVM) [20] which extends the basic generalized linear model that regresses the mean abundances $\mu_{ij}$ (for $i$-th sample and $j$-th microbe) of individual microbes against environmental covariates $x_{i}$ by incorporating latent variables $u_{i}$ as $g\left( \mu_{ij} \right)=\eta_{ij}=\alpha_{i}+\beta_{0j}+\boldsymbol{x}_{i}^{T}\boldsymbol{\beta}_{j}+\boldsymbol{u}_{i}^{T}\boldsymbol{\theta}_{j}$, where $\boldsymbol{\beta}_{j}$ are the microbe specific coefficients associated with individual covariate (a 95% confidence interval of these whether positive or negative, and not crossing 0 gives directionality with the interpretation that an increase or decrease in that particular covariate causes an increase or decrease in the abundance of the microbe), and $\boldsymbol{\theta}_{j}$ are the corresponding coefficients associated with latent variable. $\beta_{0j}$ are microbes specific intercepts, whilst $\alpha_{i}$ are optional sample effects which can either be chosen as fixed effects or random effects. To model the distribution of individual microbes, we have used Negative Binomial distribution with an additional dispersion parameter, and using log() as a link function. Additionally, the approximation to the log-likelihood is done through Variational approximation (LA) with final sets of parameters in glvmm() function being family = 'negative.binomial', method="VA", control.start=list(n.init = 8, jitter.var = 0.1), that seemed to fit well. This, we did for top 100 most abundant genera observed in our datasets. In addition, the factor loadings $\boldsymbol{\theta}_{j}$ store correlations of microbes with the residual covariance matrix $\boldsymbol{\Sigma}=\boldsymbol{\Gamma}\boldsymbol{\Gamma}^{T}$ where $\boldsymbol{\Gamma}=[\theta_{1}\ldots\theta_{m}]$ for $m$ latent variables. This residual covariance matrix gave co-occurrence relationship between microbes, that are not explained by environmental variables.

The core microbiome analysis for the stunted samples were obtained by using a 50% prevalence threshold for all OTUs according to the recommendations given in [21], and was determined using the R package microbiome [22].

In the majority of the figures displaying boxplots, pair-wise ANOVA was performed taking two categories at a time, and where significant (p ≤ 0.05), the categories were joined together by a line and the significance was plotted on top (*: 0.01 ≤ p < 0.05; **: 0.05 ≤ p< 0.001; ***: p ≤ 0.001). Furthermore, the expression level of OTUs/KOs/MetaCyc pathways are drawn using TSS+CLR normalisation (Total Sum Scaling followed by Centralised Log Ratio).

**Supplementary Table 1**. **Primers used in the study**

| Target gene | Primer sequence | Amplicon size | Reference |
| --- | --- | --- | --- |
| *Eae* | F-5’-CTGAACGGCGATTACGCGAA-3’  R-5’-CGAGACGATACGATCCAG-3’ | 482 | [23] |
| *aggR* | F-5’-GTATACACAAAAGAAGGAAGC-3’  R-5’-ACAGMTCGTCAGCATCAGC-3’ | 254 | [23] |
| *Vt* | F-5’-GAGCGAAATAATTTATATGTG-3’  R-5’-TGATGATGGCAATTCAGTAT-3’ | 518 | [23] |
| *Lt* | F-5’-GCACACGGAGCTCCTCAGTC-3'  R-5’-TCCTTCATCCTTTCAATGGCTTT-3’ | 218 | [23] |
| *sT* | F-5’GCTAAACCAGTAGAG(C)TCTTCAAAA-3’  R 5’CCCGGTACAG(A)GCAGGATTACAACA-3’ | 147 | [23] |
| *ipaH* | F-5’-CTCGGCACGTTTTAATAGTCTGG-3’  R-5’-GTGGAGAGCTGAAGTTTCTCTGC-3’ | 933 | [23] |
| *virF* | F-5’-AGCTCAGGCAATGAAACTTTGAC-3’  R-5’-TGGGCTTGATATTCCGATAAGTC-3’ | 618 | [23] |
| *daaE* | F-5’-GAACGTTGGTTAATGTGGGGTAA-3’  R-5’-TATTCACCGGTCGGTTATCAGT-3’ | 542 | [23] |
| *chuA* | F 5’ATGGTACCGGACGAACCAAC-3’  R 5’GCCGCCAGTACCAAAGACA-3’ | 288 | [24] |
| *yjaA* | F 5’ CAAACGTGAAGTGTCAGGAG-3’  R 5’AATGCGTTCCTCAACCTGTG-3’ | 211 | [24] |
| *TspE4C2* | F 5’ CACTATTCGTAAGGTCATCC-3’  R 5’ AGTTTATCGCTGCGGGTCGC-3’ | 152 | [24] |
| *ArpA* | F 5’ AACGCTATTCGCCAGCTTGC-3’  R 5’ TCTCCCCATACCGTACGCTA-3’ | 400 | [24] |

**Supplementary Figure S1**. Co-occurrence relationship between microbes recovered from the residual covariance matrix $\boldsymbol{\Sigma}$ that are not explained by the environmental covariates in the GLLVM model in Figures 6 and 7. Here, blue represent the positive correlation, and red represent the negative relationship.

**Supplementary Figure S2**. Co-occurrence relationship between microbes recovered from the residual covariance matrix $\boldsymbol{\Sigma}$ that are not explained by the environmental covariates in the GLLVM model in Figures 8 and 9. Here, blue represent the positive correlation, and red represent the negative relationship.

**Supplementary Table 2**: PERMANOVA of different distance measures against covariates considered in this study including clinical parameters.

| **Covariate** | **Bray-Curtis** | **Unweighted UniFrac** | **Weighted UniFrac** | **Functional Hierarchical Meta-storm** |
| --- | --- | --- | --- | --- |
| **Weight_kg** | *N.S* | *N.S* | *N.S* | *N.S* |
| **Vaccinated** | *N.S* | *N.S* | *N.S* | *N.S* |
| **Trimethoprim_sulfamethoxazole** | *N.S* | *N.S* | *N.S* | *N.S* |
| **Tetracycline** | *N.S* | *N.S* | *N.S* | *N.S* |
| **Source_of_drinking_water** | *N.S* | *N.S* | *N.S* | *N.S* |
| **Quality_of_diet** | *N.S* | *N.S* | *N.S* | *N.S* |
| **Phylogroup** | R2= 0.30158 (P=0.001 ***) | *R2=0.2629 (P=0.023*)* | *N.S* | R2=0.28397 (p=0.094.) |
| **Pathotypes** | *N.S* | *N.S* | *N.S* | *N.S* |
| **Nalidix_Acid** | *N.S* | *N.S* | *N.S* | *N.S* |
| **Height_cm** | *N.S* | *N.S* | *N.S* | *N.S* |
| **Gentamicin** | *N.S* | *N.S* | *N.S* | R2=0.11608 (P=0.058.) |
| **Gender** | *N.S* | *N.S* | *N.S* | *N.S* |
| **Family_type** | *N.S* | *N.S* | *N.S* | *N.S* |
| **Diagnosed_with_GIT** | *N.S* | *N.S* | *N.S* | *N.S* |
| **Ceftriaxone** | *N.S* | *N.S* | *N.S* | *N.S* |
| **Ceftazidime** | *N.S* | *N.S* | *N.S* | *N.S* |
| **Cefroflaxacin** | *N.S* | *N.S* | *N.S* | *N.S* |
| **Cefotaxime** | *N.S* | *N.S* | *N.S* | *N.S* |
| **Cefepime** | *N.S* | *N.S* | *N.S* | *N.S* |
| **Breastfed_form** | *N.S* | *N.S* | *N.S* | *N.S* |
| **Breastfed_at_birth** | *N.S* | *R2=0.05173 (p 0.048*)* | *N.S* | *N.S* |
| **Ar_conc_mg_** | *N.S* | *N.S* | *N.S* | *N.S* |
| **AP_past_3_months** | *N.S* | *N.S* | *N.S* | *N.S* |
| **Antibiotic** | *N.S* | *N.S* | *N.S* | *N.S* |
| **Ampicillin** | *N.S* | *N.S* | *N.S* | *N.S* |
| **Amikacin** | *N.S* | *N.S* | *N.S* | *N.S* |
| **Age_months** | *N.S* | *N.S* | *N.S* | *N.S* |
| **Abdominal_pain_same_day** | *N.S* | *N.S* | *N.S* | *N.S* |

**References**

1. Vonaesch P, Morien E, Andrianonimiadana L, Sanke H, Mbecko J-R, Huus KE, et al. Stunted childhood growth is associated with decompartmentalization of the gastrointestinal tract and overgrowth of oropharyngeal taxa. Proceedings of the National Academy of Sciences. 2018;115(36): E8489-E98. <https://doi.org/10.1073/pnas.1806573115>. PMID: 30126990
2. De Onis M, Blössner M, Borghi E. Prevalence and trends of stunting among pre-school children, 1990–2020. Public health nutrition. 2012;15(1):142-8. <https://doi.org/10.1017/S1368980011001315>. PMID: 21752311
3. WHO Multicentre Growth Reference Study Group (2006) WHO child growth standards based on length/height, weight and age. Acta Paediatr Suppl 450:76–85.
4. WHO Multicentre Growth Reference Study Group (2007) WHO Child Growth Standards: Head Circumference-for-Age, Arm Circumference-for-Age, Triceps Skinfold-for-Age and Subscapular Skinfold-for-Age: Methods and Development (World Health Organization, Geneva).
5. Schirmer M, Ijaz UZ, D'Amore R, Hall N, Sloan WT, Quince C. Insight into biases and sequencing errors for amplicon sequencing with the Illumina MiSeq platform. Nucleic acids research. 2015;43(6): e37-e. <https://doi.org/10.1093/nar/gku1341>. PMID: 25586220.
6. D’Amore R, Ijaz UZ, Schirmer M, Kenny JG, Gregory R, Darby AC, et al. A comprehensive benchmarking study of protocols and sequencing platforms for 16S rRNA community profiling. BMC genomics. 2016;17(1):55. <https://doi.org/10.1186/s12864-015-2194-9>. PMID: 26763898
7. Joshi N, Fass J. Sickle: A sliding-window, adaptive, quality-based trimming tool for FastQ files (Version 1.33) [Software]. 2011
8. Nikolenko SI, Korobeynikov AI, Alekseyev MA, editors. BayesHammer: Bayesian clustering for error correction in single-cell sequencing. BMC genomics; 2013: Springer. <https://doi.org/10.1186/1471-2164-14-S1-S7>. PMID: 23368723
9. Masella AP, Bartram AK, Truszkowski JM, Brown DG, Neufeld JD. PANDAseq: paired-end assembler for illumina sequences. BMC bioinformatics. 2012;13(1):31. <https://doi.org/10.1186/1471-2105-13-31>. PMID:22333067.
10. [Bolyen](https://pubmed.ncbi.nlm.nih.gov/?sort=date&size=20&term=Bolyen+E&cauthor_id=31399723) E, [Rideout](https://pubmed.ncbi.nlm.nih.gov/?sort=date&size=20&term=Rideout+JR&cauthor_id=31399723)^,^ [JR, Dillon](https://pubmed.ncbi.nlm.nih.gov/?sort=date&size=20&term=Dillon+MR&cauthor_id=31399723) MR,  [Bokulich](https://pubmed.ncbi.nlm.nih.gov/?sort=date&size=20&term=Bokulich+NA&cauthor_id=31399723) NA, [Abnet](https://pubmed.ncbi.nlm.nih.gov/?sort=date&size=20&term=Abnet+CC&cauthor_id=31399723) CC,  [Al-Ghalith](https://pubmed.ncbi.nlm.nih.gov/?sort=date&size=20&term=Al-Ghalith+GA&cauthor_id=31399723) GA. et al. Nat Biotechnol. 2019. 37(9) 1091. <https://doi>. 10.1038/s41587-019-0252-6.PMID:31399723
11. Quast C, Pruesse E, Yilmaz P, Gerken J, Schweer T, Yarza P, Peplies J, Glöckner FO. The SILVA ribosomal RNA gene database project: improved data processing and web-based tools. Nucleic Acids Res. 2013;41(Database issue):D590-6. doi: 10.1093/nar/gks1219. Epub 2012 PMID: 23193283
12. Katoh K, Standley DM. MAFFT multiple sequence alignment software version 7: improvements in performance and usability. Molecular biology and evolution. 2013;30(4):772-80. <https://doi.org/10.1093/molbev/mst010>. PMID: 23329690
13. Price OR, Williams RJ, van Egmond R, Wilkinson MJ, Whelan MJ. Predicting accurate and ecologically relevant regional scale concentrations of triclosan in rivers for use in higher-tier aquatic risk assessments. Environment international. 2010;36(6):521-6. <https://doi.org/10.1016/j.envint.2010.04.003>. PMID:20439114.
14. McMurdie PJ, Holmes S. phyloseq: an R package for reproducible interactive analysis and graphics of microbiome census data. PloS one. 2013; 8(4):e61217. <https://doi.org/10.1371/journal.pone.0061217>. PMID: 23630581.
15. Douglas, G. M., Maffei, V. J., Zaneveld, J. R., Yurgel, S. N., Brown, J. R., Taylor, C. M., ... & Langille, M. G. (2020). PICRUSt2 for prediction of metagenome functions. *Nature biotechnology*, *38*(6), 685-688.
16. Oksanen J, Blanchet FG, Friendly M, Kindt R, Legendre P, et al. Community Ecology Package Version 2.4-2 2017.
17. Zhang, Y., Jing, G., Chen, Y., Li, J., & Su, X. (2021). Hierarchical Meta-Storms enables comprehensive and rapid comparison of microbiome functional profiles on a large scale using hierarchical dissimilarity metrics and parallel computing. Bioinformatics Advances, 1(1), vbab003.
18. Vass, M., Székely, A. J., Lindström, E. S., & Langenheder, S. (2020). Using null models to compare bacterial and microeukaryotic metacommunity assembly under shifting environmental conditions. *Scientific reports*, *10*(1), 1-13.
19. Verster, A. J., & Borenstein, E. (2018). Competitive lottery-based assembly of selected clades in the human gut microbiome. *Microbiome*, *6*(1), 1-17.
20. Clarke, K. R., & Ainsworth, M. (1993). A method of linking multivariate community structure to environmental variables. *Marine Ecology-Progress Series*, *92*, 205-205.
21. Taylor, M. (2015). sinkr: A collection of functions featured on the blog’Me nugget’. R package version 1.0.
22. Niku, J., Hui, F. K., Taskinen, S., & Warton, D. I. (2019). gllvm: Fast analysis of multivariate abundance data with generalized linear latent variable models in r. Methods in Ecology and Evolution, 10(12), 2173-2182.
23. Shetty, S. A., Hugenholtz, F., Lahti, L., Smidt, H., & de Vos, W. M. (2017). Intestinal microbiome landscaping: insight in community assemblage and implications for microbial modulation strategies. *FEMS microbiology reviews*, *41*(2), 182-199.
24. Lahti, L., Shetty, S., Blake, T., & Salojarvi, J. (2017). Microbiome R package. Tools Microbiome Anal R.
25. Vidal M, Kruger E, Durán C, Lagos R, Levine M, Prado V, Toro C, Vidal R. Single multiplex PCR assay to identify simultaneously the six categories of diarrheagenic Escherichia coli associated with enteric infections. J Clin Microbiol. 2005 Oct;43(10):5362-5. doi: 10.1128/JCM.43.10.5362-5365.2005. PMID: 16208019; PMCID: PMC1248459.
26. Clermont O, Christenson JK, Denamur E, Gordon DM. The Clermont Escherichia coli phylo-typing method revisited: improvement of specificity and detection of new phylo-groups. Environ Microbiol Rep. 2013 Feb;5(1):58-65. doi: 10.1111/1758-2229.12019. Epub 2012 Dec 24. PMID: 23757131
